# Engineered fluidic systems to understand lymphatic cancer metastasis

**DOI:** 10.1063/1.5133970

**Published:** 2020-01-28

**Authors:** Joshua D. Greenlee, Michael R. King

**Affiliations:** Department of Biomedical Engineering, Vanderbilt University, Nashville, Tennessee 37235, USA

## Abstract

The majority of all cancers metastasize initially through the lymphatic system. Despite this, the mechanisms of lymphogenous metastasis remain poorly understood and understudied compared to hematogenous metastasis. Over the past few decades, microfluidic devices have been used to model pathophysiological processes and drug interactions in numerous contexts. These devices carry many advantages over traditional 2D *in vitro* systems, allowing for better replication of *in vivo* microenvironments. This review highlights prominent fluidic devices used to model the stages of cancer metastasis via the lymphatic system, specifically within lymphangiogenesis, vessel permeability, tumor cell chemotaxis, transendothelial migration, lymphatic circulation, and micrometastases within the lymph nodes. In addition, we present perspectives for the future roles that microfluidics might play within these settings and beyond.

## INTRODUCTION: THE RISE OF MICROFLUIDICS

The discrepancies between scientific data gathered *in vitro* and *in vivo* vs clinical settings suggest that new models are warranted to recapitulate human pathophysiological processes.^1^ For years, there has existed a challenge in the field of bioengineering and drug discovery concerning the effectiveness of 2D cell cultures to model human physiology and drug interactions observed clinically.^1–4^ Two-dimensional static cultures remain the standard for cellular biology; yet, these models lack physiological relevance and have often proven ineffective as clinical predictors due to the dilute and ineffective recapitulation of the cellular microenvironment.^4,5^ While *in vivo* models remain necessary to assess drug interactions in the preclinical setting, the average success rate of translation from animal models to clinical cancer trials is less than 8%.^6^ Aside from being “lost in translation,” animal models raise ethical concerns and are problematic when using human cells due to host–immune cell interactions.^2,6^

The combination of these shortcomings has pushed research into the direction of using 3D and microfluidic platforms to recapitulate the physical and chemical microenvironments seen *in vivo*, while providing the means to precisely control and visualize cellular interactions in a high-throughput manner. Microfluidics employs the use of small channels on the scale of tens to hundreds of micrometers in diameter to process minute fluid volumes.^7^ Their small size allows for limited cell numbers or reagents, which are often expensive or low in quantity. Furthermore, these devices can more accurately model *in vivo* architectures through integration of 3D extracellular matrix (ECM) components.^2,3,8^ The spatiotemporal control of these devices has allowed researchers to study specific cellular interactions in a more precise and controlled manner. Microfluidics is also advantageous as it can be fabricated to incorporate small working distances to allow for high-resolution, real time imaging.

The usage of biologically compatible material substrates from molds allows for high-throughput production of devices and subsequent analysis. A large majority of microfluidic devices for biological application use soft lithography techniques, which include fabricating a master “stamp” from a photocurable polymer such as SU-8.^9^ This master can be used to imprint features into elastomeric materials, such as polydimethylsiloxane (PDMS), with high resolution. PDMS is widely used in microfluidics since it is easy to handle, can be purposed in diverse applications, is economically viable, ideal for imaging due to its optical properties, and, most importantly, is biologically inert.^10^ While the field of microfluidics has advanced tremendously in terms of applications, the versatility of replication molding with PDMS meant that new fabrication techniques have lagged behind. Other means of fabrication often require advanced equipment and are not economically feasible at a small scale for research purposes, use materials that do not translate well with biological applications, or lack the high-resolution capabilities inherent with soft lithography.^9^ However, certain applications may require more intricate fabrication techniques, such as micromachining, 3D printing, or dry etching. Table I illustrates the ubiquity of PDMS and photolithography in the field of microfluidics for biomedical research.

**TABLE I.** Microfluidic devices to model the stages of lymphogenous metastasis. Primary references are listed first, followed by supporting literature describing fabrication methodology where applicable.

Application	Device summary	Substrate	Fabrication method	Major finding	Cell lines	Year	Reference
Lymphangiogenesis and LEC barrier function	Nine chamber radial flow device to model capillary morphogenesis under interstitial flow with applications in TME modeling	PDMS with fibrin gels	PMMA micromachining	Interstitial flow is vital for lymphatic morphogenesis, migration and proper molecular gradients *in vitro*	HMVEC (human dermal Microvascular endothelial cells) (lymphatic and blood), human dermal fibroblasts, B16-F10 (murine melanoma)	2010	61
	Mimicking drainage function seen in lymphatic microvasculature	PDMS with fibrin gels	SU-8 photolithography	Drainage is required to preserve vascular stability and perfusion rates within fibrin scaffolds	HMVEC	2013	64 and 65
	Microcirculation model featuring both blood and lymphatic vessels for the purpose of examining vascular permeability	PDMS/PET membrane with fibronectin	SU-8 photolithography	Vascular permeability in a coculture LEC and BEC system mimicked responses seen *in vivo*	HMVEC (lymphatic and blood)	2015	66 and 67
	Mimicking LEC sprouting seen *in vivo* using physical and biochemical cues	PDMS with fibrin gels	SU-8 photolithography	Interstitial flow-initiated outgrowth of lymphatic sprouts toward upstream of the flow while suppressing downstream-directed sprouting	HMVEC (lymphatic), NHLF (normal human lung fibroblasts)	2016	62
	Modeling lymphangiogenesis and angiogenesis simultaneously within tumor microenvironment	PDMS with collagen-fibrin gels	SU-8 photolithography	Mimicked simultaneous angiogenesis and lymphangiogenesis of the TME using interactions of tumor cells with cellular and noncellular components	HUVEC (human umbilical vein endothelial cells), HMVEC (lymphatic), primary fibroblasts, SKOV3 (human ovarian adenocarcinoma), MKN-74 (human stomach adenocarcinoma), and SW620 (human colorectal adenocarcinoma)	2017	63
LEC/tumor cell crosstalk	Chemotaxis of tumor cells toward lymphatics via CCR7 signaling within a modified Boyden chamber	Modified Boyden chamber with Matrigel	…	Physiological levels of IF can enhance tumor cell migration in the direction of flow via CCR7 autocrine signaling	HMVEC (lymphatic), MCF10A (human breast epithelial), MCF7 (human breast adenocarcinoma), ZR75-1 (human breast carcinoma), and MDA-MB-435 (human melanoma)	2007	76
	Modeling crosstalk between LECs and cancer cells via VEGF-C and CCR7 signaling in a modified Boyden chamber	Modified Boyden chamber with collagen I	…	VEGF-C acts in an autocrine fashion to increase tumor invasiveness by increasing the proteolytic activity and motility of tumor cells	HEUVEC, HMVEC (lymphatic), MDA-MB-435S	2009	77
	Pressure gradient across collagen gels creates interstitial flow that influences tumor cell migration	PDMS with collagen I	SU-8 photolithography	Interstitial flow creates competing mechanisms of tumor migration downstream (CCR7 dependent) and upstream (CCR7 independent)	MDA-MB-231, MDA-MB-435	2011	78 and 79
	Modeling extravascular migration of tumor cells along lymphatics via 3D confined cell migration	PDMS with collagen IV	SU-8 photolithography	Subpopulations of cells showed sustained migratory potential despite treatment with Taxol chemotherapeutics	MDA-MB 231, H1650 (lung adenocarcinoma), H446 Lung carcinoma, PC3 (prostate adenocarcinoma), LnCaP (prostate carcinoma), U-87MG (glioblastoma), HT-29 (colorectal adenocarcinoma)	2013	83
	IFN-DC migration and interactions with cancer cells within 3D tumor environments	PDMS with collagen I	SU-8 photolithography	CXCR4/CCL12 axis guides IFN-DC toward apoptotic tumor cells for antigen uptake	Primary IFN-DCs (IFN-alpha Dendritic cells), SW620	2017	88
	First *ex vivo* crosstalk system via secreted factors between lymph node and tumor samples	PDMS	SU-8 photolithography	Lymph node slices cocultured with tumor slices appeared more immunosuppressed than those cocultured with healthy tissue	Murine LN and tumor slices	2019	87
Transendothelial migration	Microgaps force cell deformation to study effects on transmigration potential	PDMS with Matrigel	SU-8 photolithography	Extravasation potential was significantly affected by endothelial lining	HMVEC, HepG2 (hepatocellular carcinoma), HeLa (cervical adenocarcinoma), MDA-MB-435	2007	98 and 99
	Tumor cell interactions with endothelial cell barrier function	PDMS with hydrogel	SU-8 photolithography	Macrophage-secreted TNF-α induces endothelium permeability and tumor cell intravasation	HT1080, MDA-MB-231, RAW264.7 (macrophages)	2012	93
	Tumor cell interactions with artificial microvasculature under physiological shear	PDMS with collagen I	Aluminum replication molding	Cancer cells show biased growth toward vessels but do not contact the endothelium for transendothelial migration	MDA-MB-231, HT-1080, HUVEC, HMVEC	2014	94
	Perfusable metastasis chip with microposts allowing for endothelial cell monolayer formation for angiogenesis and intravasation	PDMS with fibrinogen	SU-8 photolithography	TNF-α treated EC monolayers demonstrated increased permeability and subsequent intravasation	MDA-MB-231	2014	70
	Cancer cell transmigration under transmural and interstitial flow across LEC monolayers	Somoss Watershed XC11122	High-resolution stereolithography	Luminal and transmural flow upregulate tumor cell transmigration, partially through LEC CCL21 upregulation	HMVEC (lymphatic), MDA-MB-231	2015	101
	Simple transwell device to study mechanisms of tumor cell TEM in LECs	Transwell plates	…	Lymphangiogenic peptide adrenomedullin facilitates TEM by promoting cancer cell binding to LECs and gap junction coupling	HMVEC (lymphatic), SK-MEL-2 (human melanoma), MCF7	2015	103
	Simple transwell device with interstitial flow to study leukocyte and cancer cell transmigration	Transwell plates	…	Cancer cells prefer basal to apical transmigration in LECs compared to BECs	MCF7, MDA-MB-231, HMVEC, mouse primary dermal lymphatic endothelial cells, SVEC4-10 (mouse endothelial)	2017	102
	Microfluidic channels separated by porous membrane lined with primary endothelial cell monolayer	PDMS with collagen I	Replication molding	Cancer cells adhere to endothelium under flow, but transmigration was not observed	HMVEC, H838 (non-small cell lung cancer), SK-Mel-28 (human melanoma)	2018	95
Lymphatic circulating tumor cells	Flow chamber to study tumor cell behaviors under lymphatic shear conditions	Parallel plate laminar flow chamber coated with collagen	…	Colorectal cancer cells remained attached, proliferative and alive under lymphatic shear conditions	RKO (human colorectal carcinoma), HCT-8 (colorectal adenocarcinoma)	2007	106
	Cone-and-plate viscometer to apply hematogenous shear conditions to CTCs	Cone-and-plate viscometer	…	Cancer cells are sensitized to cytotoxic ligand TRAIL under hematogenous shear conditions	COLO205 (colorectal adenocarcinoma), PC3 (prostate adenocarcinoma)	2013	107 and 108
	Migration channels with choke points to mimic lymphatic capillary geometry and confinement	PDMS with collagen I	Soft lithography	(MAPK) family member, p38γ knock out cells show decreased motility through tighter choke point geometries	MDA-MB-231	2015	111
	Microcavities that model architecture of micrometastases in the lymph node	PDMS (gas expansion molding)	Deep reactive ion etching	Engineered natural killer cells can eradicate LN micrometastases in 3D microcavities	LnCAP, COLO205 MDA-MB-231	2014	112 and 113

Replication of tissue microenvironments within microfluidic devices has allowed the modeling of complex physiological process systems in “organ on a chip” devices.^11–16^ Meanwhile, the incorporation of multiple organs on a chip in one integrated device can be used for the scaling of “microHumans” to study complex anatomical interactions and systemic drug toxicity.^17,18^ Although initially intended to bridge the gaps between 2D *in vitro* studies and *in vivo* work, 3D fluidic models are evolving to study pathologies and drug interactions directly in patient-specific devices.^2,19–23^ These technological advances have made it possible to study specific diseases, including cancer within a microfluidic device.^1,2,8,12,24–26^ The applications for microfluidic devices are evolving and emerging in the field of cancer research, both from a biological perspective of understanding roles of immune and stromal cells to a translational perspective of investigating the efficacy of therapeutics in preclinical models.

## THE ROLE OF LYMPHATICS IN CANCER METASTASIS

Approximately 90% of all cancer related deaths are attributed to metastasis.^27^ Despite its high morbidity, cancer metastasis is a very inefficient process in which less than 0.1% of circulating tumor cells (CTCs) will actually go on to colonize and form macrometastases.^28^ The metastatic cascade is composed of several sequential steps, each of which selects for a specific cellular phenotype that is able to overcome inhospitable environments. First, cancer and stromal cells in the primary tumor secrete proangiogenic factors such as VEGF to promote tumor microvasculature networks of both blood and lymphatic vessels. Tumor cells then undergo an epithelial to mesenchymal transition (EMT) that promotes cell motility through the loss of cell–cell adhesion proteins such as E-cadherin and β-catenin.^29^ Motile cells will then migrate and invade the basement membrane of the nearby vasculature through both physical (high intratumoral pressures) and chemical (chemokine gradients) cues.^28^ Cells may enter the hematogenous or lymphatic circulation via transendothelial migration (TEM) from the tissue parenchyma into nearby blood or lymphatic vessels, respectively.^27^ In the case of lymphatic intravasation, tumor cells will drain into collecting lymphatic vessels, eventually emptying into the sentinel or “tumor draining lymph nodes” (TDLN).^30^ Successful migration to the sentinel lymph nodes provides cells with a direct route to systemic lymph nodes and the bloodstream via the thoracic duct and subclavian vein.^31,32^ Once in circulation, cells can undergo extravasation, followed by a mesenchymal to epithelial transition (MET) and colonization of distant organs.^27^ As suggested from Paget's “seed and soil” hypothesis, tumor cells will have genetic and phenotypic advantages to promote seeding in specific organs over others.^33,34^ Just as a seed needs proper nutrients to grow, tumor cell proliferation and survival is highly dependent upon the microenvironment where CTC extravasation occurs. This sequential model of metastasis is well studied but has proven to be an oversimplification in many cancer models and in clinical observation.^31,35^

It is estimated that 80% of carcinomas and melanomas metastasize via lymphatics.^30^ Despite the fact that the majority of all human cancers metastasize initially via the lymphatic system, the mechanisms of lymphogenous metastasis remain poorly understood and understudied compared to that of hematogenous metastasis.^30,36^ There are many factors that help determine which metastatic route a cell will take.^37^ Typically, metastatic subpopulations will develop mutational burdens, which may be preferential for one mode over another. For instance, CCR7+ tumor cells will preferentially traffic toward CCL21 secreted by lymphatic endothelial cells (LECs), promoting initial metastasis through the lymphatic system. This signaling axis is typically used in CCR7+ dendritic cells (DCs) trafficking into lymph nodes, while T-cells expressing CCR7 follow gradients toward increasing CCL19 secreted within the thymus and lymph nodes.^38^ Additionally, CCL21 can be upregulated by VEGF-C/VEGFR-3 signaling, which has been shown to be highly expressed in primary tumors and tumor-derived lymphatic neovasculature.^31^ By harnessing these signaling mechanisms employed by immune cells to traffic toward LECs for antigen presentation, cancer cells experience directed migration toward the lymphatic circulation.^31,36^ Physical and mechanical forces have also been shown to play a role in lymphatic homing. High interstitial pressures within solid tumors promote interstitial flow (IF) toward the periphery of the tumor where lymphatic vessels are concentrated.^39^ Interstitial flow has been shown to promote vascular remodeling and even promote tumor cell invasion via autologous chemotaxis toward lymphatic vessels along the tumor periphery. Furthermore, secretion of specific prolymphangiogenic factors can favor lymphatics vs blood.^32^ For example, upregulated VEGF-C and VEGF-D secretion by cancer cells have been shown to induce preferential lymphatic metastasis via LEC VEGFR-2 and VEGFR-3, whereas blood endothelial cell (BEC) angiogenesis prefers VEGF-A/VEGFR-1 signaling.

Lymphatic capillaries lack pericytes and tight interendothelial junctions typically seen in blood vessels.^31,40^ The leaky nature of lymphatic vessels facilitates tumor cell intravasation via transendothelial migration, promoting initial metastasis. Likewise, the lower fluid shear environment within lymphatic vessels (in the range of 0.4 dyne/cm^2^ with surges between 4 and 12 dyne/cm^2^)^41,42^ compared to blood vasculature (upward of 30 dyne/cm^2^ in arteries)^43,44^ increases the likelihood of cell survival in transit.^37^ Mounting evidence exists suggesting that lymphatics may also play a role in curbing antitumor immune responses.^45^ For example, initial lymphatic vessels formed as a result of tumor-induced lymphangiogenesis exhibit upregulated expression of the immune checkpoint ligand programmed cell death ligand 1 (PD-L1), which induces CD8+ T-cell anergy upon tumor associated antigen (TAA) presentation via MHC class 1.^31,36,46^ In addition, tumor-induced LECs have been shown to prevent dendritic cell maturation, increase T-cell tolerization, and inhibit proliferation of T-cells that have been stimulated by proinflammatory cytokines.^36,47,48^

Despite the increasing evidence for the roles of lymphatics in promoting cancer progression and dissemination, there are innate characteristics of lymphatics that promote antitumor immunity. For instance, the presence of lymphatic networks at the primary tumor are important for TAA trafficking to immune cells to evoke a robust T-cell response.^45^ In addition, lymphatic vessel density in solid tumors strongly correlates with the quantity of infiltrating cytotoxic CD8+ T-cells, promoting “hot” vs “cold” tumors in patients.^49,50^

The involvement of lymphatics in cancer dissemination extends into the clinic. The presence of metastases in the sentinel lymph nodes of cancer patients is used as a basis for establishing tumor staging, predicting patient prognosis and formulating treatment strategies.^51^ Axillary and sentinel lymph node biopsies in melanoma and breast cancer patients have proven to be fundamental for assessing the aggressiveness and extent of disease.^52,53^ In fact, sentinel lymph node (LN) biopsies will often reveal metastatic spread before detection by traditional imaging modalities such as positron emission tomography/computed tomography (PET-CT) or before the presence of blood-borne CTCs.^51^ The clinical ramifications of this on the degree of lymphatic involvement in metastasis demonstrates the importance of the role of the lymphatic system in cancer.

The complex roles of lymphatics in metastatic progression are not yet fully understood. What is apparent, however, is that better understanding of the interplay between tumor cells, their microenvironment, and the lymphatic system during metastasis is vital to the discovery of new therapeutics to exploit tumor cell weaknesses. The tight control of physical and chemical stimuli, high-throughput nature, high-resolution capacity, and physiologically relevant architecture of microfluidics provide excellent means for better understanding these intricate interactions.

## MICROFLUIDIC MODELS OF LYMPHOGENEOUS METASTASIS

### Lymphangiogenesis and LEC barrier function

Lymphatic vasculature is comprised of initial lymphatic vessels and collecting vessels, which function to prevent the accumulation of fluid, termed edema, in tissue.^31,54^ Additionally, these vessels function to transport pathogens, antigens, and antigen presenting cells (APCs) from tissues toward immune cells residing within the lymph nodes. Initial lymphatic vessels, also known as lymphatic capillaries, range from 35 to 70 *μ*m in diameter and absorb interstitial fluids, facilitated by pressure gradients between the interstitium and vessel lumen.^55^ The lymph then drains into downstream collecting vessels where unidirectional valves and smooth muscle contractions facilitate transport into the draining lymph nodes. Lymphangiogenesis is the analog to angiogenesis, the process in which lymphatic endothelial cells sprout to create new vasculature off of existing vessels. Tumor-induced lymphangiogenesis is characterized by VEGF-C or VEGF-D overexpression in tumors, which has been correlated with an increase in lymph node metastasis and high morbidity in patients.^56–58^ Lymphatic vessels have a high permeability due to discontinuous interendothelial junctions and a sparse surrounding basement membrane.^32^ While this facilitates immune cell intravasation and extravasation, it makes vessels susceptible to metastatic tumor cells as well.

**FIG. 1.**
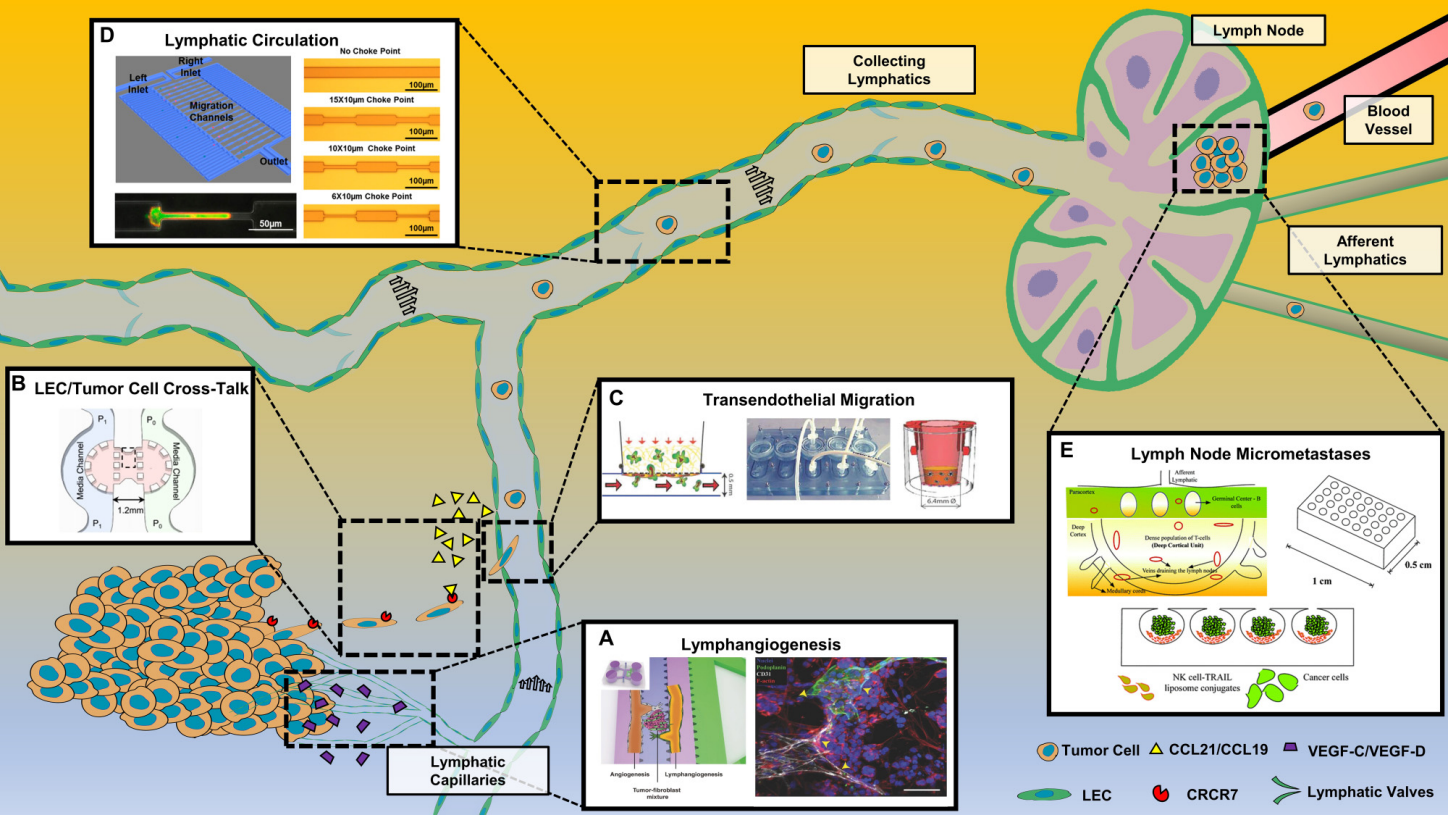
Current microfluidic models to study the mechanisms of lymphatic cancer metastasis. Representative devices used to study (a) lymphangiogenesis [reproduced with permission from Chung *et al*., Adv. Healthc. Mater. **6**, 1700196 (2017). Copyright 2017 John Wiley and Sons], (b) crosstalk between LECs and tumor cells [reproduced with permission from Polacheck *et al*., Proc. Natl. Acad. Sci. U.S.A. **108**, 11115 (2011). Copyright 2011 National Academy of Sciences], (c) Transendothelial migration [reproduced with permission from Pisano *et al*., Integr. Biol. (Camb.) **7**, 525. Copyright 2015 Oxford University Press], (d) CTCs in lymphatic circulation [reproduced with permission from Chen *et al.*, Sci. Rep. **5**, 9980 (2015); Copyright 2015 Author(s), licensed under a Creative Commons Attribution Springer Nature License],^111^ and (e) Formation of lymph node micrometastases [reproduced with permission from Chandrasekaran *et al*., Lab Chip **14**, 118 (2014). Copyright 2014 Royal Society of Chemistry].

Interstitial flow has been shown to be an important regulator of lymphangiogenesis *in vitro* and *in vivo*.^59,60^ To model this, the Swartz Lab created a multichambered, high-throughput flow device capable of replicating interstitial flow pressures through a 3D extracellular matrix.^61^ This PDMS device allowed live imaging of morphogenesis of lymphatic and blood endothelial cells while incorporating tumor microenvironment (TME) components with cocultures of tumor cells and fibroblasts. A related study by Kim *et al*. also investigated the roles of IF on lymphangiogenesis, discovering directionality of lymphatic sprouting was flow dependent.^62^ Interstitial flow-induced upstream lymphatic sprouting and suppressed downstream sprouting via LEC polarization. This PDMS device consisted of a center 3D fibrin channel containing LECs and microposts, surrounded by two flow channels and a 3D fibroblast culture. The same group later adapted the design to study the complex networks of lymphatic vessels in coculture with BECs, fibroblasts, and cancer cells in a 3D tumor microenvironment model, as shown in Fig. 1(a).^63^ This is the only known study to mimic angiogenesis and lymphangiogenesis simultaneously in one 3D microfluidic platform.

Interstitial flow is also key in preserving endothelial barrier function in lymphatic neovasculature.^39,60^ A study by Wong *et al*. used a new device to study endothelial cell perfusion to demonstrate the importance of lymphatic drainage in preserving vascular stability.^64,65^ This device in particular has the capability of being repurposed by introducing coculture with cancer cells to study endothelial cell permeability and transendothelial migration under lymphatic drainage conditions. More recently, a device by Sato *et al*. investigated vascular permeability within cocultures of LECs and BECs.^66^ The PDMS device consisted of two microfluidic channels where LECs and BECs were cultured back-to-back, separated by a fibronectin coated polyethylene terephthalate (PET) membrane.^67^ Their findings demonstrated that physiological flow conditions enhanced cell–cell junctions and recapitulated microvascular architecture seen *in vivo*.^66^

While angiogenesis is a well characterized process that has been incorporated into many microfluidic devices,^68–73^ the process of lymphangiogenesis is less well understood. The described microfluidic devices have demonstrated advances in understanding the roles of lymphatic vessel sprouting, morphogenesis, and permeability in the context of the tumor microenvironment. In particular, within the last few years there has been emphasis on the fabrication of more complex systems that model lymphangiogenesis in parallel with angiogenesis. While multiple devices have been employed to study lymphatic microvasculature function, few have incorporated components to model lymphangiogenesis in the context of the tumor microenvironment. Modifying or reapplying these devices to represent tumor-induced vascular remodeling will be instrumental in the future. The field is mature from a device design standpoint, but there remain many opportunities within existing devices for further studies, especially regarding drug modulation of lymphangiogenesis and LEC barrier function. Currently, there are no FDA-approved compounds to prevent tumor-induced lymphangiogenesis, supporting the use of such microfluidic devices to study novel drug candidates before human trials.^74^

### Lymphatic-induced migration and tumor crosstalk

The likelihood of successful metastatic dissemination is contingent upon a tumor cell's migratory potential.^75^ Cancer cells in the primary tumor undergo EMT characterized by a loss of adhesion proteins like E-cadherin and the upregulation of vimentin.^29^ These cancer cells resemble a cancer stem cell phenotype and morphology, including a decrease in proliferation and increase in migratory capacity. As previously alluded to, these cancer cells are able to harness the same chemotactic mechanisms typically employed by immune cells to migrate toward and into initial lymphatics.^31,36^ Meanwhile, high intratumoral pressures direct interstitial flows toward the tumor periphery into peritumoral lymphatic vessels.^39^ High interstitial flows can cause fibroblast contraction and collagen fiber alignment, along with tumor stiffening. This will increase tumor invasiveness, as cells will more easily migrate through aligned collagen fibers. When taken together, all these mechanisms promote tumor cell migration away from the primary tumor and toward peritumoral vasculature.

The Swartz Lab has pioneered microfluidic modeling of tumor cell chemotaxis toward lymphatics. In one study, Shields *et al*. used a modified 3D Boyden chamber, consisting of a tumor cell culture in 3D ECM with LECs cultured on the underside of the chamber.^76^ By introducing interstitial flows of just 0.2 *μ*m/s through the 3D ECM in the absence of LECs, cancer cell migration was enhanced via autologous CCR7 signaling. This novel finding first suggested that IF not only promoted directed migration through physical mechanisms but also through autocrine signaling where a chemotactic gradient naturally forms at the leading edge of the cell. When cultured with LECs, paracrine CCL21 secretion enhanced CCR7 signaling and offered a complementary role for lymphatic-directed chemotaxis. A separate study from the Swartz Lab built upon this observation in a similarly modified Boyden chamber device. Issa *et al*. demonstrated that tumor cell VEGF-C enhanced LEC CCL21 secretion through VEGFR-3 signaling, thereby enhancing tumor cell proteolysis and migration toward LECs.^77^

Polacheck *et al*. created a two-channel PDMS device separated by a collagen interface in which a pressure difference between channels drove interstitial flow through the device, as shown in Fig. 1(b).^78,79^ This study further supported the previously demonstrated phenomena of autologous CCR7 chemotaxis downstream of interstitial flow. However, when blocking CCR7 signaling, migration was directed upstream of flow, hypothesized to be linked to flow-induced tension in integrins via phosphorylation of focal adhesion kinase (FAK).

Tumor cells under confinement show preferential migration in paths of least resistance through trajectories created by leader cells,^80^ collagen fiber alignment,^81^ or on the periphery of preexisting lymphatic and blood vessels.^82^ Irimia and Toner created a high-throughput model of cancer cell migration under confinement using collagen-filled, cell-sized microchannels in 96 well plates.^83^ When treated with paclitaxel chemotherapy, overall cell migratory potential of MDA-MB-231 cells was significantly decreased. However, subpopulations of cells proved resistant to migratory inhibition and showed sustained migration in the presence of high concentrations of drug.

Tumor cells have been shown to create tolerization of immune cells prior to inhabiting the TDLN, creating a premetastatic niche that can be ideal for tumor cell seeding.^30,84^ Moreover, technological advancement in the way of *ex vivo* tissue cultures has enabled more translational studies of drug–tumor interactions and personalized medicine.^85,86^ Recently, Shim *et al*. created the first *ex vivo* crosstalk system via secreted factors between lymph node and tumor slices. They designed a multilayer PDMS device with integrated pumps to recirculate supernatant between tumor and LN tissue under physiological interstitial flow conditions.^87^ Their findings demonstrated that LN tissues cultured with tumor tissue contained immunosuppressed T-cell populations, as characterized by decreases in IFN-γ secretion, supporting established *in vivo* findings.

Since cancer cell and immune cell migration employ the use of the same chemokine signaling axes, it is important to understand how migration is modulated by the presence of drugs or immunotherapies. Parlato *et al*. created a PDMS device with an immune chamber and tumor chamber separated by confined connecting chambers to demonstrate the mechanisms behind IFN-α-conditioned DC migration toward tumor cells.^88^ Their results demonstrated that the CXCR4/CCL12 axis guides dendritic cells toward apoptotic cancer cells leading to TAA phagocytosis and cross-presentation to naïve T-cells. This study focused on immune cell migration upon treatment of tumor cells, but the device could straightforwardly be repurposed to examine simultaneous immune cell and cancer cell-directed migration in the presence or absence of drugs.

Overall, this has been the most studied stage in lymphatic metastasis using microfluidics. Precise control over device characteristics such as collagen density (and consequential stiffness), flow profiles, pressure gradients, chemotactic gradients, and channel architectures make microfluidic devices well suited for modeling tumor migration and lymphatic crosstalk. However, to date, no device yet exists to study LEC-induced chemotaxis of cancer cells simultaneously in the presence of immune cells. Most current systems are binary, only comprised of cancer cell lines in culture with LECs, or in the case of the previously described device, only address mechanisms of immune cell trafficking. Modeling the roles of LEC directed migration with lymphatics and cancer cells in tandem will be important for understanding drug interactions to prevent off target effects on immune cells. Moreover, the addition of immune cells will provide insights into symbiotic relationships between immune cells and cancer cells during lymphatic-directed migration. Furthermore, the inclusion of other cell types such as cancer associated fibroblasts (CAFs) will be key in elucidating the complex roles these cell types have during migration.^89^ More recent studies incorporating primary samples and *ex vivo* tissue samples are gaining traction due to their translational relevance.^85,86^ It is expected that this trend will continue, specifically with applications in personalized medicine. One could imagine the reapplication of the aforementioned Shim *et al*. *ex vivo* device,^87^ with patient derived slices of an excised tumor and LN samples to study patient immune tolerization and immunotherapy efficacy.

### Transendothelial migration through lymphatic endothelium

A cancer cell's ability to disseminate to other organs is fully dependent upon its ability to enter the lymphatic or hematogenous circulation.^28^ Intravasation is the process in which cancer cells invade the basement membrane of the vasculature and then enter the circulation through a process known as transendothelial migration (TEM). There are two modes of TEM: paracellular (through endothelial cell–cell junctions) or transcellular (through endothelial cell bodies).^90^ TEM in the context of cancer intravasation or extravasation has been studied in a range of microfluidic devices.^2,3,91,92^ However, until recently, these studies were carried out predominantly with BECs in the context of blood vessel intravasation and extravasation.

The Kamm lab created a successful device that set a precedent for the study of endothelial barrier function in the context of tumor metastasis.^93^ Their PDMS device consisted of two independent channels where endothelial cells and tumor cells were seeded, separated by a 3D ECM hydrogel region. Permeability of BEC monolayers cocultured with macrophages and subsequent transmigratory potential of HT1080 fibrosarcoma cells were quantified. This study was instrumental in elucidating the role of macrophage-secreted TNF-α in endothelial monolayer permeability and tumor intravasation potential. Building on this, another group created artificial microvasculature from cylindrical channels lined with endothelial cells to study cancer cell migration and intravasation into perfusable vessels.^94^ Another system was used to study extravasation, where a two-chamber PDMS device was split with a porous membrane containing an endothelial monolayer.^95^ Cancer cells were perfused through the top chamber, while the bottom chamber contained a reservoir to collect any extravasated cancer cells. While the cancer cells adhered to the endothelium, no transendothelial migration was observed within the short time frame of cell rolling. These are just some of the many current microfluidic platforms used to study cancer cell migration through the blood endothelium.^3,91,92,95–97^ Multiple devices have been created to study TEM through vascular endothelium in concert with other metastatic processes. Lee *et al*. created a “metastasis chip” that modeled both angiogenesis and subsequent intravasation of MDA-MB-231 cells together in one platform.^70^ Likewise, Chaw *et al*. created a multistep device where cells underwent deformation through 10 *μ*m trenches before passing through an endothelial monolayer.^98,99^ The latter has applications in studying cell confinement through vessel contraction and subsequent lymphatic extravasation, an understudied phenomena.

More recently, microfluidic devices have been fabricated for the purpose of investigating the role of the lymphatic endothelium in TEM. Increasing emphasis on the role of lymphatics in initial metastasis along with the innate differences between blood and lymphatic endothelium has motivated such studies. For instance, unlike vascular endothelial monolayers, lymphatic monolayers are characterized by having increased permeability, an incomplete or absent basement membrane, and sparse, overlapping intercellular junctions.^100^ The Swartz Lab pioneered one of the first devices of cancer cell transmigration in LECs. They fabricated a five-channel microfluidic chamber that was designed to deliver both luminal and transmural flow to LEC monolayers, as shown in Fig. 1(c).^101^ Tumor cells in a 3D extracellular matrix were cultured above a membrane containing the monolayer. This device demonstrated that luminal, interstitial, and transmural flow promoted intravasation of MDA-MB-231 cells. The device was validated by demonstrating that luminal flow augmented LEC expressed CCL21 to drive cancer cell migration. Xiong *et al*. created a simplified version that used transwell inserts coated with an LEC monolayer to study vectorial migration and intravasation of immune cells and breast cancer cell lines.^102^ This more recent model was designed to be more readily accessible and easier to use for other research labs to study TEM. A similar system using transwell inserts was used by Karpinich and Caron to study tumor cell interaction with lymphatic endothelium.^103^ Their study demonstrated that the peptide adrenomedullin promotes coupling of cancer cells to LEC gap junctions and facilitates heterocellular communications to induce transendothelial migration.

Microfluidic platforms are excellent tools for studying transendothelial migration of cancer cells, largely due to the ease of visualization via live cell imaging. In addition, precise control of endothelial monolayers more closely mimics endothelial barrier function observed *in vivo*. Due to known differences between the lymphatic and blood endothelium, there exists a need to understand the different roles they play in relation to cancer cell intravasation. The majority of established hematogenous intravasation and extravasation devices could be readily modified to study LEC barrier function as well. Although straightforward in principle, inherent differences between blood and lymphatic endothelium will require these repurposed devices to be thoroughly screened and calibrated with LECs. If successfully implemented, future studies may examine differences in the transmigratory potential of cancer cells between BECs and LECs within the same device, potentially revealing subpopulations of phenotypes that are prone to lymphatic vs hematogenous TEM. In addition, cancer cell extravasation through lymphatics is poorly understood and few *in vitro* or *in vivo* models exist to study this phenomenon. Modifying existing extravasation devices by culturing LECs instead of BECs will allow for modeling cancer cell rolling, arrest within the lymph node, and extravasation from lymphatics into nearby or distal tissues.

### Lymphatic circulating tumor cells

Once cancer cells transmigrate through the endothelium and enter the lymphatic circulation, they are subject to a unique physical and chemical environment.^28,31,36,37,54^ There are many differences in the rheology and flow dynamics between lymphogenous and hematogenous circulation. With the absence of red blood cells and platelets, the viscosity of lymph and interstitial fluid can be two- to fourfold less than that of blood.^104,105^ Initial lymphatic vessels that have a low Reynolds number flow within the Stokes flow regime, and in the largest vessels draining into the thoracic duct, the flow remains laminar.^54^ This deviates from arterial blood flow, which is mostly laminar but can become turbulent in larger arteries. Overall, the higher shear rates within the blood flow make CTC survival inauspicious compared to those within lymphatics.

Early efforts to model shear effects of lymphatics on metastatic tumor cells included the use of parallel plate flow chambers, as demonstrated in a previous study with colorectal cancer cell lines.^106^ In that study, a constant shear stress of 1.2 dyne/cm^2^ was applied to cancer cells while cell proliferation, spreading, and apoptosis were quantified. In two separate studies, our lab modeled dynamic shear on CTC's in human blood using a cone-and-plate viscometer.^107,108^ These studies demonstrated that physiological shear stress can sensitize cancer cells to TNF-α related apoptosis inducing ligand (TRAIL) via the activation of mechanosensitive ion channels.^109^ Similar to parallel plate flow chambers, a cone-and-plate viscometer is widely available, easy to use, and readily adaptable to model a variety of different physiological shear processes, making it suitable for studying cancer cells in lymphatic transit.

The lymphatic system utilizes both extrinsic and intrinsic phasic pumping mechanisms from the surrounding lymphatic muscle to produce the pulsatile flow of lymph from tissue.^41,54^ The contractile properties of the lymphatics can create confined architectures for cancer cells, augmenting cell motility, proliferation, and survival via the process of mechanotransduction.^110^ Chen *et al*. created a migration device with choke points ranging from 6 to 30 *μ*m to model metastasis through lymphatic capillaries.^111^ This PDMS device, as shown in Fig. 1(d), consisted of two separated serpentine channels, one loaded with cells and the other with chemotactic agents, separated by straight migration channels with constricted choke points of various diameters. Using MDA-MB-231 cells, cell migration through tight choke points was revealed to be dependent on Map Kinase family member p38γ.

Despite the high concentrations of immune cells surveilling the lymphatics, cancer cells are often able to seed within the sentinel lymph nodes and form micrometastases.^30^ Often small and clinically undetectable without a lymph node biopsy, these cancer cells can reside and remain senescent for years while evading immune detection. To mimic this, our group created a PDMS microcavity device that recapitulated the architecture of the lymph node, as shown in Fig. 1(e).^112^ The device was fabricated using deep reactive ion etching in silicon, followed by gas expansion molding in PDMS to create spherical microbubbles.^113^ Natural killer cells were cocultured with cancer cells, modeling interactions between micrometastases, immune cells, and therapeutics. Although this study was only conducted under static conditions, the same device was used in a separate study under a continuously perfused flow to culture 3D spheroids.^114^

Currently, there exist flow devices that can be applied to the study of lymphatic and hemodynamic shear on migratory cancer cells. Both parallel plate flow chambers and cone-and-plate viscometers are easily adaptable and widely available devices to study such phenomena. Despite this, surprisingly few studies exist to study cancer cell transit in lymphatic circulation. This represents an important research opportunity to make these devices more physiologically relevant. This may include culturing LEC monolayers on the inner walls of a device while perfusing intraluminal lymphatic flow to CTCs. Furthermore, the creation of a device that allows for vessel dilation and constriction via smooth muscle, or artificially via transmural pressure, would better replicate the lymphatic behavior experienced *in vivo*. These physiological conditions may be modeled after similar devices that use whole artery or vein segments *ex vivo*.^115,116^ Meanwhile, there is a need for more devices to model cancer cell seeding and senescence within the tumor draining lymph nodes, specifically devices that incorporate immune cell interactions with cancer cells. There currently are multiple “lymph node on a chip” devices existing outside of the applications of cancer.^117–119^ Although outside of the scope of this review, incorporating cancer cells within lymph node on chip microenvironments may elucidate the mechanisms behind cancer cell seeding and survival.

## CONCLUSIONS AND FUTURE PERSPECTIVES

Despite their great potential and versatility, microfluidic devices have not been fully harnessed to study the intricacies of lymphogenous metastasis. While there are an abundance of microfluidic devices studying metastasis in the context of the bloodstream, few devices exist that incorporate lymphatics as part of or the focus of their model. This is surprising since the majority of all cancers metastasize via lymphatics and the mechanisms of lymphogenous metastasis are in many ways as poorly understood as that of hematogenous metastasis.^30^ Given that the infrastructure of the aforementioned devices are widely adaptable, we propose that progress within the field will mostly come from new applications of previously developed systems. A germane initial step would be to recreate previous studies, such as those modeling angiogenesis or TEM into the blood vessels but replacing blood microvasculature with lymphatic microvasculature. Even more ideal would be the incorporation of blood vessels and lymphatics within the same device, similar to that described by Sato *et al*.^66^ This could be instrumental in elucidating how cells differentiate between lymphogenous vs hematogenous metastasis, while characterizing subpopulations that are predisposed to one mode over another. Moreover, combining multiple stages within one device, as done by Lee *et al*., who studied both angiogenesis and intravasation,^70^ will be useful to determine how different metastatic steps affect one another. Fabrication of these all-in-one “lymphatic metastasis on a chip” devices will advance the field toward a device capable of modeling the entire metastatic cascade within one platform.

As previously mentioned, there may be difficulties with replacing BECs with LECs in existing devices. Although both are endothelial cells and carry out similar functions, they have distinct transcriptional profiles, which make them unique in culture.^120^ For example, BECs appear to be more reliant on ECM interactions for proper functionality, indicating that ECM components in existing devices with endothelial monolayers may need to be tailored to suit LEC culture. Additionally, lymphatic endothelium is known to have relatively looser interendothelial junctions, which could pose challenges for culturing uniform monolayers within devices.^31,32^ Incorporating LECs into existing devices will require careful observation and calibration to ensure physiological relevance, especially when adapting features such as flow profiles, cell densities, and ECM concentrations.

Immune cells play complex roles in relation to cancer development, and as such, numerous microfluidic devices exist to study these interactions.^2,117,119,121–123^ Surprisingly, these devices tend to look exclusively at cancer–immune cell interactions strictly within the tumor microenvironment, not in relation to their roles during metastasis. Cancer cells trafficking toward and into the lymph nodes are likely to interact in some capacity with both adaptive and innate immune cells, further warranting their inclusion within these microfluidic systems. Investigating the roles of the immune system will be key to not only understanding how cancer cells can leverage these interactions but also for exploiting cancer cell weaknesses with immunotherapies.

For more translational studies to exist, microfluidic devices must become more user-friendly and compatible for use in the clinical setting. This includes the integration of automated image processing, routine sample processing, and minimalization of complex system components to allow for the analysis to be carried out to completion within hospital laboratories. Meanwhile, reproducibility of such devices will be necessary for widespread implementation. The first step in the inclusion of clinical microfluidic devices would be validating drug toxicology and biological phenomena observed *in vivo* and in humans. This includes examining drug interactions of approved FDA compounds that have extensive clinical data and comparing those same interactions within relevant microfluidic devices for validation purposes. With regard to lymphatic devices, testing an FDA-approved compound such as sorafenib, which has been shown to interfere with LEC expressed VEGFR-2 and VEGFR-3 and has been approved for the treatment of metastatic renal cell carcinoma,^124^ within a lymphangiogenesis device would be applicable for validating modeling capabilities. In addition, testing well characterized checkpoint inhibitors such as PD-L1 targeting antibody atezolizumab to demonstrate blockage of checkpoint signaling by LECs would provide insights into the mechanisms behind an LEC targeted therapy.^125^

From a research perspective, new platforms to promote collaborations between biologists and engineers are warranted. This framework will in turn promote the fabrication of devices to answer pressing questions in the field of biology, rather than attempting to fit biological applications within preexisting, incompatible devices. A 2014 study by Sackmann *et al*. estimated that only 6% of all microfluidic devices are published in biology and medicine journals.^126^ Interdisciplinary work within this field will be crucial for improved biological modeling and drug discovery.
